# T Follicular Helper Cells and B Cell Dysfunction in Aging and HIV-1 Infection

**DOI:** 10.3389/fimmu.2017.01380

**Published:** 2017-10-23

**Authors:** Suresh Pallikkuth, Lesley de Armas, Stefano Rinaldi, Savita Pahwa

**Affiliations:** ^1^Department of Microbiology & Immunology, University of Miami Miller School of Medicine, Miami, FL, United States

**Keywords:** T follicular helper cells and HIV, T follicular helper cells and immunity, HIV and aging, T follicular helper cells and influenza vaccine, T follicular helper cells in aging and HIV

## Abstract

T follicular helper (Tfh) cells are a subset of CD4 T cells that provide critical signals to antigen-primed B cells in germinal centers to undergo proliferation, isotype switching, and somatic hypermutation to generate long-lived plasma cells and memory B cells during an immune response. The quantity and quality of Tfh cells therefore must be tightly controlled to prevent immune dysfunction in the form of autoimmunity and, on the other hand, immune deficiency. Both Tfh and B cell perturbations appear during HIV infection resulting in impaired antibody responses to vaccines such as seasonal trivalent influenza vaccine, also seen in biologic aging. Although many of the HIV-associated defects improve with antiretroviral therapy (ART), excess immune activation and antigen-specific B and T cell responses including Tfh function are still impaired in virologically controlled HIV-infected persons on ART. Interestingly, HIV infected individuals experience increased risk of age-associated pathologies. This review will discuss Tfh and B cell dysfunction in HIV infection and highlight the impact of chronic HIV infection and aging on Tfh–B cell interactions.

## Introduction

Chronic infectious diseases, such as HIV infection, and the biological process of aging are known to impact humoral immune responses to vaccination and infection (1–5). The issue of aging during HIV infection has gained importance due to the success of antiretroviral therapy (ART) that can lead to near normal life expectancy and is resulting in increasing the numbers of aging HIV-infected people (3, 6, 7). Older HIV-uninfected individuals in the general population, especially those >80 years develop immune senescence, a term signifying immune defects affecting multiple cell types, characterized by quantitative reduction in hematopoietic stem cells, thymic involution with reduced naive cells and accumulation of effector and memory cell subsets with narrow TCR repertoires with low clonality, and reduced CD4:CD8 T cell ratio (8–11). Memory T cells tend to lose expression of CD28 and their antigen-specific responses are impaired (12). In addition, profound B cell alterations occur in biologic aging characterized by a reduction of the naive B cell pool and qualitative impairment of their function along with reduced vaccine induced immune responses (13–22). Concurrently, increased inflammation coined by the term inflamm-aging (21, 23) occurs with increased C-reactive protein (CRP), D-dimer, IL-6, and TNFα that correlate with occurance of age-associated diseases.

Immunologic changes similar to biologic aging have been described in HIV infection, including accelerated immune senescence and inflammation, with increased IL-6, CRP, and D-dimer (24–26) despite virologic suppression with ART and have been attributed to persistent immune activation (25, 27–29). Cellular markers of immune senescence, including low CD4:CD8 ratio and higher frequencies of CD57 + CD28− CD4 and CD8 T cells are prominent especially in those who initiate ART at lower CD4 counts. Based upon epigenetic changes, age of HIV inflected people is approximately 5 years greater (and more without viral suppression) than uninfected people (30, 31) of the same chronologic age. They manifest increased risk for non-AIDS morbidity and mortality, including neurocognitive decline, cardiovascular disease, kidney disease, and cancer (32). Because of the associated immune deficiency in both biologic aging and HIV infection, and the aging of HIV-infected population, it is important to determine how the immune systems in HIV-infected and -uninfected differ and to delineate the underlying mechanisms which could lead to therapeutic interventions. This review will focus on cellular basis of vaccine responses in the context of T follicular helper (Tfh) cells and their interaction with B cells, how these cells are affected by HIV infection and finally discuss recent findings on the impact of aging in HIV-infected and -uninfected persons using response to influenza vaccine as a readout of immune competence.

### Tfh Cells in Lymph Node (LN) and Periphery

T follicular helper cells are a specialized subset of CD4 T cells in lymphoid organs that express the transcription factor B cell CLL/lymphoma 6 (Bcl-6), with high surface expression of programed death receptor 1 (PD-1) and CXC chemokine receptor 5 (CXCR5) [reviewed in Refs. (33–37)]. During an immune response Tfh cells provide critical signals to antigen-experienced B cells in germinal centers (GCs) to undergo proliferation, isotype switching, and somatic hypermutation (SHM) in order to generate long-lived plasma cells and memory B cells through cellular interaction and cross-signaling for antibody production [reviewed in Refs. (37–39)]. Tfh cell differentiation requires dendritic cell (DC) priming of naive antigen-specific CD4 T cells followed by the interaction with B cells resulting in upregulation of costimulatory molecules such as inducible costimulator (ICOS) and CD40 ligand (CD40L) and secretion of cytokines IL-21 and IL-4 that play a critical role for the ensuing B cell response [reviewed in Refs. (33, 34, 39)].

Because of the difficulties in studying lymphoid tissue in humans, the field has increasingly relied on a circulating subset of memory CD4 T cells that partially resemble LN Tfh cells and have been designated as peripheral Tfh (pTfh) (40–47). The pTfh cells display a memory phenotype and are characterized by expression of CXCR5, the B cell follicle homing molecule, and by secretion of IL-21 during interactions with B cells (42, 48). Unlike LN Tfh cells, pTfh cells express only moderate levels of PD-1 and Bcl-6 but are similar in their ability to upregulate costimulatory molecules such as ICOS and CD40L upon antigen stimulation (42, 49–52). More recently, based on the surface expression of CXCR3, CCR6 and CXCR4 Tfh cells have been further characterized as Th1 (CXCR3 + CCR4 − CCR6−), Th2 (CXCR3 − CCR4 + CCR6−), and Th17 (CXCR3 − CCR4 − CCR6+) memory CD4 T helper subtypes (42, 53, 54), indicative of reveals the heterogeneous nature of pTfh cells with respect to phenotypic, functional and transcription factor profiles (42, 54). It is now widely considered that a balance of pTfh subsets is important for maintaining healthy immune function.

### Tfh, B cells, and HIV infection

T follicular helper cells are highly permissive to HIV becoming readily infected by follicular DC that transport infectious virions into lymphoid organs. Tfh cells are now considered as major reservoirs of transcriptionally silent integrated HIV genomes (55–58). In non-human primates, chronic infection with simian immunodeficiency virus (SIV) is associated with an expansion of Tfh cells within GC (59, 60), along with increase in numbers of B cells in LN, spleen, and gut tissues of rhesus macaques (60–63). Early initiation of ART can rapidly control the virus replication but not the early lymphoid activation, thereby increasing the risk of infection of Tfh and magnitude of viral reservoir (64). Contrary to the expansion of GC Tfh cells seen in chronic HIV/SIV infection (59, 60), we and others have reported a significant loss of circulating pTfh cells in chronic viremic HIV-infected subjects compared to HIV-uninfected persons (65, 66); 12 months of ART incorporating Raltegravir resulted in increased frequencies of pTfh cells (66). However, pTfh cells from HIV^+^ virologically suppressed patients on ART exhibit functional impairment in their ability to provide adequate B cell help in a number of systems (41, 67–69).

In chronic HIV infection, B cells exhibit immune dysfunction and altered B cell subset distribution, with a shift in resting memory (RM) B cells to an activated state with expression of activation markers such as CD71, CD80, and CD86 (70, 71). There is also an increase in inflammatory B cell subsets referred to as double negative (DN: CD27 − IgD − B cells) and tissue-like memory B cells (15, 72–75). ART-mediated viral suppression restores many of the B cell defects, especially when initiated during the acute phase of infection (76). However, reduced frequencies of RM B cells, elevated DN B cells, as well as chronic immune activation persist (31, 71, 77–79).

### Vaccine-Induced Antibody Responses During HIV Infection

In healthy states, antibody responses to T-dependent antigens are generated in GCs within lymphoid tissue when antigen-primed B and T cells engage in interactions to promote B cell differentiation, SHM, and class switch recombination to develop into memory B cells and plasma cells (80–83). Studies in humans and animal models indicate that HIV infection affects the GC reaction, increases immune activation/exhaustion of lymphocytes, and results qualitative deficiency of Tfh and B cell function (57, 59–61, 69). These defects altogether lead to increased susceptibility to vaccine-preventable diseases (84, 85). Studies focusing on pTfh cells have been informative for understanding the phenotypic complexity within the Tfh subset and for determining the relationship between Tfh and B cells in immunological outcomes [reviewed in Ref. (86)].

Influenza vaccine studies have provided a valuable model system to analyze the immune system in vaccine induced antibody responses (87). We initiated such studies in virally suppressed HIV^+^ adults on ART during the 2009/H1N1 pandemic influenza outbreak (43, 88, 89). Following monovalent H1N1 vaccination, vaccinees were classified as vaccine responders (VRs) if postvaccination hemagglutination inhibition (HAI) serum H1N1 Ab titer was 1:40 or more and exhibited a 4-fold increase, from baseline titer, and those who did not meet these criteria were classified as vaccine non-responders (VNRs). In study participants, administration of the vaccine resulted in VR status only in 50% HIV^+^, compared to all age matched healthy controls. In the HIV + VR and VNR, prevaccination CD4 and CD8 T cell counts, B cell frequencies, and plasma HIV RNA were similar, but phenotypic and qualitative immunological differences were identified. In VR, there was upregulation of IL-21R in B cells that correlated with plasmablasts and memory B cell responses post-vaccination (89), together with an expansion of pTfh cells with secretion of IL-21 and CXCL-13 in H1N1-stimulated PBMC culture supernatants. In coculture experiments, pTfh supported HIN1-stimulated IgG production by autologous B cells (43). More recent findings point to the ability to perform qualitative assessment of pTfh/CD4 T cells and B cells prior to immunization in previously vaccinated HIV^+^ children and young adults (90, 91). Examples of such assessments include (i) *ex vivo* stimulation with H1N1 resulting in induction of CXCR5 mRNA and protein in CD4 T cells and (ii) induction of *IL21* gene in pTfh cells. These antigen-specific prevaccination measures strongly associated with H1N1-specific B cell responses by ELISPOT at postvaccination (91). Interestingly, CD4 T cells from VNR exhibit increased expression of *IL2* and *STAT5* genes, which are known to antagonize pTfh function (92). Our main findings of pTfh and B cells in relation to vaccine responses are summarized in Table 1. Other vaccine studies have shown associations between pTfh expansion and phenotype with vaccine response. Expansion of HIV-specific PD-1 + ICOS + pTfh correlated with vaccine-specific serum IgG after booster immunization in three different human HIV vaccine trials (93). Expression of ICOS, PD-1, CD38, and IL-21 in pTfh subsets have been useful for evaluating the influenza vaccine response in HIV-infected and -uninfected adults in other studies as well (50, 87, 93–95). Studies with Ebola vaccine (rVSV-ZEB OV) demonstrated that CXCR5 + PD-1 + pTfh correlated with expansion of plasmablasts (96). Taken together, these studies support the concept that both quality and quantity of pTfh cells are important determinants for the outcome of vaccine response in HIV infection.

**Table 1:** Signature immunological changes in pTfh and B cells in vaccine responders (VRs) following influenza vaccine at TO (baseline), T1 (7 days), and T2 (4 weeks).

**Changes in pTfh cell compartment in vaccine responders** Antigen induced IL-21 gene expression at TO Expansion of pTfh at T1, T2 Ag-stimulated intracellular IL-21 production in pTfh at T2 “Help” to autologous B cells for H1N1-specific IgG production and B cell differentiation in pTfh plus B cell cocultures at T2
**B cell changes in vaccine responders** Increase in frequencies of plasmablasts at T1 Increase in spontaneous H1N1-specific ASC at T1 Increase in memory B cells and switch memory at T2 Upregulation of IL-21R on total B and memory B cells at T2 Increase in TACI expression on total B and memory B cells at T2 Downregulation of BAFT-R expression on total B and memory B cells at T2
**PBMC culture sups/plasma findings in vaccine responders** Production of IL-21 and CXCL13 in H1N1-stimulated culture sups with increases in plasma IL-21 Increase in plasma BAFF and APRIL levels

*pTfh, peripheral T follicular helper; PBMCs, peripheral blood mononuclear cells; Ab, antibody; BAPF-R, B cell activating factor receptor; APRIL, a proliferation inducing ligand; CXCL13, C-X-C motif chemokine ligand 13; ASCs, antibody secreting cells*.

### Tfh Cells and B Cells in HIV and Aging

Our group has been interested in the question of immune function of aging HIV^+^ individuals who are well controlled on ART, the extent to which it resembles biologic aging of HIV^−^ individuals, and implications of aging with HIV infection. Earlier pilot studies in virologically suppressed postmenopausal women as representative of an aging population established the persistence of inflammation and gut microbial translocation and detrimental role of underlying immune activation on influenza vaccine responses that were associated with quantitative and qualitative deficiencies of pTfh cells (45, 97, 98). Our studies showed lower H1N1 influenza antibody titers in HIV-infected women compared to HIV-uninfected women at prevaccination. Following vaccination, magnitude of antibody responses and frequency of study participants achieving seroprotective titers were lower in HIV^+^ than in HIV^−^ women. Frequencies of pTfh cells at postvaccination correlated with memory B cell function and H1N1 antibody titers. Antibody responses postvaccination were inversely correlated with inflammatory cytokine TNFα in plasma and with markers of cellular immune activation (CD38 and HLA-DR) on CD4 T cells, including pTfh subset, indicating an adverse influence of baseline immune activation and inflammation on vaccine induced antibody response in older age.

To examine the role of age and HIV infection further, we are engaged in a large ongoing study (99, 100) in virologically suppressed HIV^+^ and HIV^−^ adults grouped by age as young (<40 years), middle aged (40–59 years), and old (≥60 years). Following seasonal trivalent influenza vaccine (TIV), magnitude of Ab titers against each vaccine strain were found to be lower in old age compared to others, regardless of HIV status. Baseline titers in seroprotective range were higher in HIV^+^ but the frequency of VR was lower in HIV^+^ than HIV^−^. Interestingly the young HIV^+^ showed maximum variance from HIV^−^ and more rapid decay in titer after peak at 28 days postvaccination. In statistical analysis somewhat surprisingly effect of age rather than HIV dominated the impaired immune response observed in old persons (age > 60 years), whereas HIV clearly had a strong effect on immunity at younger ages (99, 100).

We examined phenotypic characteristics of T and B cells in this group of participants prior to vaccination. T cell phenotypic analysis revealed a core signature of aging comprised of decreasing naive T cells and a loss of CD38 expression on CD4 and CD8 T cells. Frequencies of activated CD4 T cells (and not CD8 T cells) identified by coexpression of HLA-DR and CD38, as well as expression of PD-1, ICOS, and Ki-67 were higher in HIV^+^ participants compared to HIV^−^ participants. Increases in activation markers previously associated with aging such as ICOS (87) were already evident in young HIV^+^ compared to young HIV^−^, indicative of HIV causing a state of premature immune senescence. Predictive modeling to determine the key T cell variables most closely associated with vaccine response revealed pTfh as an important biomarker. In HIV^−^, baseline pTfh frequency was positively associated with vaccine response, while in HIV^+^ expression of multiple activation markers on pTfh (including PD-1) was negatively associated with vaccine response (99).

**Figure 1:**
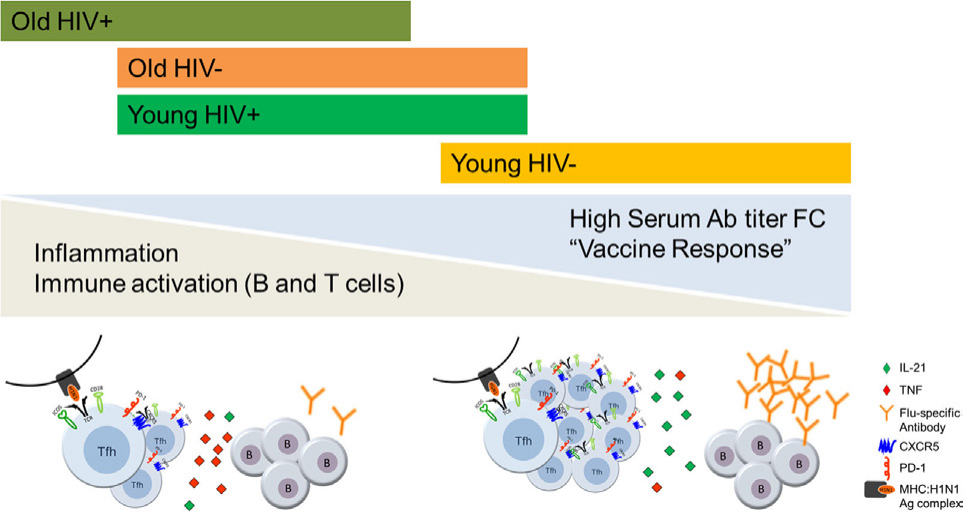
The effects of aging and HIV infection on T follicular helper (Tfh):B cell responses to influenza vaccination. Persistent inflammation and immune activation of CD4 T cells and B cells negatively influence the outcome of influenza vaccine response in antiretroviral therapy (ART)-treated HIV-infected virologically suppressed individuals through impairing the Tfh and B cell functions. HIV induced premature Immunosenescence further advanced immune dysfunction which is more evident in the young HIV^+^ individuals.

Prevaccination status of B cells also revealed perturbations as evidenced by alteration in markers of activation, exhaustion and immune regulation and were more prevalent in young HIV^+^ than in young HIV^−^ (100). HIV infection in younger adults exhibited similarities with biological aging resulting in alterations in B cell phenotypic and functional characteristics similar to those observed in older HIV^−^ individuals but underlying mechanisms appear to be distinct from that associated with biological aging (100). For example, the interaction between T and B cells through the PD-1:PD-L1 signaling pathway is involved only in HIV induced impairment of B cell function (101). These results provide the basis for immune correlates of premature aging in HIV^+^, even with prolonged ART-induced virological suppression (Figure 1). Additional mechanistic studies to understand the cellular basis of immunological impairments in pTfh and B cells in aging and HIV infection are currently ongoing in our laboratory.

Other factors that could influence the influenza vaccine response in aging also need consideration. Data from literature suggest that vaccine-induced immune responses are considerably influenced by demographic variables such as age, sex, ethnicity, and race (102–105). Many studies indicate that aged females consistently have higher antibody responses and increased vaccine efficacy to influenza vaccines than males [reviewed in Refs. (106, 107)]. Sex differences in HAI antibody titers to either the standard-dose or high-dose influenza vaccine are apparent, in which antibody responses are higher in older females than in males (108, 109). A role played by male hormone testosterone in lowering the immune response has been proposed (109, 110). There is growing interest in how latent cytomegalovirus (CMV) infections impact the outcome of vaccination [reviewed in Ref. (111)]. In young adults, CMV infection is associated with elevated antibody responses to influenza vaccines. In aged individuals, CMV seropositivity is associated with chronic inflammation and lower antibody responses to influenza vaccines (112, 113). However, lack of association between CMV status and influenza response in elderly population has also been reported (114). Thus the overall impact of CMV infection on influenza vaccine responsiveness remains controversial. A direct link between CMV seropositivity with increased risk of influenza illness in vaccinated older adults has not been reported in either HIV-infected or healthy individuals. Moreover, the influence of gender and CMV infection status on the cellular basis of immune impairment involving pTfh and B cell compartments are not been studied in aging and HIV infection. In aged mice, CD4^+^ and CD8^+^ T cells express several inhibitory receptor molecules, including PD-1, LAG-3, CTLA-4, and KLRG1 (115, 116) that could interfere with the immune response to vaccination. Prolonged expression of inhibitory molecules is a well-known feature of T cell exhaustion in chronic viral infections and exhausted T cells have also been identified in different viral infections, such as HIV and hepatitis A and B virus in humans [reviewed in Refs. (117–120)]. However, further studies are warranted to elucidate the significance of T cell exhaustion in HIV infection in the context of aging and its influence on vaccine induced immune response through regulation of pTfh and B cell function.

## Conclusions and Future Perspectives

Development of a protective antibody response to vaccine or infection is important for the control or eradication of many pathogenic infections. Efficient Tfh–B cell interactions are required for regulating B cell differentiation toward the development of high affinity antibodies. Immune mechanisms underlying the regulation of Tfh–B cell interactions at the inductive sites of the immune response are an active area of immunology research. Several studies have highlighted the qualitative and quantitative impairment of Tfh compartment and their subsequent impact on humoral arm of immune response in treated HIV infection (43, 45, 67, 87, 94, 98). Since HIV-infected people are aging, research on the cumulative impact of premature and physiological immune senescence on immune function in HIV infection is of great importance. Our work underscores the adverse effect of inflammation, a cardinal feature associated with biologic aging and chronic HIV infection, on immune response to vaccination and functional impairment of Tfh and B cells as a consequence of persistent immune activation.

Recent advances in the field of immune checkpoint inhibitor-based immunotherapeutic approaches in cancer immunology have highlighted the importance of cell to cell interactions on immune function. Many aspects of checkpoint molecule-based regulation of humoral immune response on Tfh and B cell interactions at the GC are not known. Trials employing checkpoint inhibitors in HIV infection will need to ensure that improved Tfh–B cell interactions not associated with autoimmunity. Immune checkpoints are negative regulators of T cell activation, T cell proliferation and effector functions and inhibiting immune checkpoints could influence and disrupt the resting status of latently infected cells and reverse latency with increase in HIV replication within GC (121). Future studies are needed to explore combination approaches targeting immune checkpoint molecules and costimulatory signaling pathways during an immune response to understand the coregulation of immunity by these molecules in the GC reaction. The ultimate goal should be to establish strategies to improve the immune function at inductive sites. Interventions aimed at reducing chronic inflammation and immune activation along with immunomodulatory approaches may improve response to vaccines in aging HIV^+^ individuals.

## Author Contributions

All authors listed have made a substantial, direct, and intellectual contribution to the work and approved it for publication.

## Conflict of Interest Statement

The authors declare that the research was conducted in the absence of any commercial or financial relationships that could be construed as a potential conflict of interest.

## References

[1] Dubrow R Silverberg MJ Park LS Crothers K Justice AC. HIV infection, aging, and immune function: implications for cancer risk and prevention. Curr Opin Oncol (2012) 24(5):506–16. 10.1097/CCO.0b013e328355e131 22759737 PMC3695478

[2] Reber AJ Chirkova T Kim JH Cao W Biber R Shay DK Immunosenescence and challenges of vaccination against influenza in the aging population. Aging Dis (2012) 3(1):68–90. 22500272 PMC3320806

[3] Wing EJ. HIV and aging. Int J Infect Dis (2016) 53:61–8. 10.1016/j.ijid.2016.10.004 27756678

[4] Gianella S Letendre S. Cytomegalovirus and HIV: a dangerous pas de deux. J Infect Dis (2016) 214(Suppl 2):S67–74. 10.1093/infdis/jiw217 27625433 PMC5021239

[5] Franceschi C Capri M Monti D Giunta S Olivieri F Sevini F Inflammaging and anti-inflammaging: a systemic perspective on aging and longevity emerged from studies in humans. Mech Ageing Dev (2007) 128(1):92–105. 10.1016/j.mad.2006.11.016 17116321

[6] Deeks SG Verdin E McCune JM Immunosenescence and HIV. Curr Opin Immunol (2012) 24(4):501–6. 10.1016/j.coi.2012.05.004 22658763

[7] Desai S Landay A. Early immune senescence in HIV disease. Curr HIV/AIDS Rep (2010) 7(1):4–10. 10.1007/s11904-009-0038-4 20425052 PMC3739442

[8] Naylor K Li G Vallejo AN Lee WW Koetz K Bryl E The influence of age on T cell generation and TCR diversity. J Immunol (2005) 174(11):7446–52. 10.4049/jimmunol.174.11.7446 15905594

[9] Pulko V Davies JS Martinez C Lanteri MC Busch MP Diamond MS Human memory T cells with a naive phenotype accumulate with aging and respond to persistent viruses. Nat Immunol (2016) 17(8):966–75. 10.1038/ni.3483 27270402 PMC4955715

[10] Ferguson FG Wikby A Maxson P Olsson J Johansson B. Immune parameters in a longitudinal study of a very old population of Swedish people: a comparison between survivors and nonsurvivors. J Gerontol A Biol Sci Med Sci (1995) 50(6):B378–82. 10.1093/gerona/50A.6.B378 7583794

[11] Yoshida K Cologne JB Cordova K Misumi M Yamaoka M Kyoizumi S Aging-related changes in human T-cell repertoire over 20years delineated by deep sequencing of peripheral T-cell receptors. Exp Gerontol (2017) 96:29–37. 10.1016/j.exger.2017.05.015 28535950

[12] Weng NP Akbar AN Goronzy J. CD28(-) T cells: their role in the age-associated decline of immune function. Trends Immunol (2009) 30(7):306–12. 10.1016/j.it.2009.03.013 19540809 PMC2801888

[13] Khurana S Frasca D Blomberg B Golding H. AID activity in B cells strongly correlates with polyclonal antibody affinity maturation in-vivo following pandemic 2009-H1N1 vaccination in humans. PLoS Pathog (2012) 8(9):e1002920. 10.1371/journal.ppat.1002920 23028320 PMC3441753

[14] Frasca D Diaz A Romero M Blomberg BB Human peripheral late/exhausted memory B cells express a senescent-associated secretory phenotype and preferentially utilize metabolic signaling pathways. Exp Gerontol (2016) 87(Pt A):113–20. 10.1016/j.exger.2016.12.001 27931848

[15] Colonna-Romano G Bulati M Aquino A Pellicano M Vitello S Lio D A double-negative (IgD-CD27-) B cell population is increased in the peripheral blood of elderly people. Mech Ageing Dev (2009) 130(10):681–90. 10.1016/j.mad.2009.08.003 19698733

[16] Frasca D Blomberg BB. Effects of aging on B cell function. Curr Opin Immunol (2009) 21(4):425–30. 10.1016/j.coi.2009.06.001 19608393 PMC2853364

[17] Frasca D Diaz A Romero M Landin AM Blomberg BB. Age effects on B cells and humoral immunity in humans. Ageing Res Rev (2011) 10(3):330–5. 10.1016/j.arr.2010.08.004 20728581 PMC3040253

[18] Gibson KL Wu YC Barnett Y Duggan O Vaughan R Kondeatis E B-cell diversity decreases in old age and is correlated with poor health status. Aging Cell (2009) 8(1):18–25. 10.1111/j.1474-9726.2008.00443.x 18986373 PMC2667647

[19] Troutaud D Drouet M Decourt C Le Morvan C Cogne M. Age-related alterations of somatic hypermutation and CDR3 lengths in human Vkappa4-expressing B lymphocytes. Immunology (1999) 97(2):197–203. 10.1046/j.1365-2567.1999.00779.x 10447732 PMC2326838

[20] van Dijk-Hard I Soderstrom I Feld S Holmberg D Lundkvist I. Age-related impaired affinity maturation and differential D-JH gene usage in human VH6-expressing B lymphocytes from healthy individuals. Eur J Immunol (1997) 27(6):1381–6. 10.1002/eji.1830270613 9209488

[21] Franceschi C Bonafe M Valensin S Olivieri F De Luca M Ottaviani E Inflamm-aging. an evolutionary perspective on immunosenescence. Ann N Y Acad Sci (2000) 908:244–54. 10.1111/j.1749-6632.2000.tb06651.x 10911963

[22] Weinberger B Herndler-Brandstetter D Schwanninger A Weiskopf D Grubeck-Loebenstein B. Biology of immune responses to vaccines in elderly persons. Clin Infect Dis (2008) 46(7):1078–84. 10.1086/529197 18444828

[23] Harris TB Ferrucci L Tracy RP Corti MC Wacholder S Ettinger WH Jr Associations of elevated interleukin-6 and C-reactive protein levels with mortality in the elderly. Am J Med (1999) 106(5):506–12. 10.1016/S0002-9343(99)00066-2 10335721

[24] Robbins GK Spritzler JG Chan ES Asmuth DM Gandhi RT Rodriguez BA Incomplete reconstitution of T cell subsets on combination antiretroviral therapy in the AIDS Clinical Trials Group protocol 384. Clin Infect Dis (2009) 48(3):350–61. 10.1086/595888 19123865 PMC2676920

[25] Appay V Sauce D Assessing immune aging in HIV-infected patients. Virulence (2017) 8(5):529–38. 10.1080/21505594.2016.1195536 27310730 PMC5538339

[26] Kaplan-Lewis E Aberg JA Lee M. Aging with HIV in the ART era. Semin Diagn Pathol (2017) 34(4):384–97. 10.1053/j.semdp.2017.04.002 28552209

[27] Angelovich TA Hearps AC Maisa A Martin GE Lichtfuss GF Cheng WJ Viremic and virologically suppressed HIV infection increases age-related changes to monocyte activation equivalent to 12 and 4 years of aging, respectively. J Acqui Immune Defic Syndr (2015) 69(1):11–7. 10.1097/QAI.0000000000000559 25647525

[28] Cobos Jimenez V Wit FW Joerink M Maurer I Harskamp AM Schouten J T-cell activation independently associates with immune senescence in HIV-infected recipients of long-term antiretroviral treatment. J Infect Dis (2016) 214(2):216–25. 10.1093/infdis/jiw146 27073222 PMC8445638

[29] Nasi M De Biasi S Gibellini L Bianchini E Pecorini S Bacca V Ageing and inflammation in patients with HIV infection. Clin Exp Immunol (2017) 187(1):44–52. 10.1111/cei.12814 27198731 PMC5167025

[30] Horvath S Levine AJ. HIV-1 infection accelerates age according to the epigenetic clock. J Infect Dis (2015) 212(10):1563–73. 10.1093/infdis/jiv277 25969563 PMC4621253

[31] Gross AM Jaeger PA Kreisberg JF Licon K Jepsen KL Khosroheidari M Methylome-wide analysis of chronic HIV infection reveals five-year increase in biological age and epigenetic targeting of HLA. Mol Cell (2016) 62(2):157–68. 10.1016/j.molcel.2016.03.019 27105112 PMC4995115

[32] Deeks SG HIV infection, inflammation, immunosenescence, and aging. Annu Rev Med (2011) 62:141–55. 10.1146/annurev-med-042909-093756 21090961 PMC3759035

[33] Crotty S. Follicular helper CD4 T cells (TFH). Annu Rev Immunol (2011) 29:621–63. 10.1146/annurev-immunol-031210-101400 21314428

[34] Crotty S. The 1-1-1 fallacy. Immunol Rev (2012) 247(1):133–42. 10.1111/j.1600-065X.2012.01117.x 22500837

[35] Fazilleau N Mark L McHeyzer-Williams LJ McHeyzer-Williams MG. Follicular helper T cells: lineage and location. Immunity (2009) 30(3):324–35. 10.1016/j.immuni.2009.03.003 19303387 PMC2731675

[36] Qi H. T follicular helper cells in space-time. Nat Rev Immunol (2016) 16(10):612–25. 10.1038/nri.2016.94 27573485

[37] Crotty S. T follicular helper cell differentiation, function, and roles in disease. Immunity (2014) 41(4):529–42. 10.1016/j.immuni.2014.10.004 25367570 PMC4223692

[38] Crotty S. A brief history of T cell help to B cells. Nat Rev Immunol (2015) 15(3):185–9. 10.1038/nri3803 25677493 PMC4414089

[39] Webb LMC Linterman MA. Signals that drive T follicular helper cell formation. Immunology (2017) 152(2):185–94. 10.1111/imm.12778 28628194 PMC5588773

[40] Chevalier N Jarrossay D Ho E Avery DT Ma CS Yu D CXCR5 expressing human central memory CD4 T cells and their relevance for humoral immune responses. J Immunol (2011) 186(10):5556–68. 10.4049/jimmunol.1002828 21471443

[41] Locci M Havenar-Daughton C Landais E Wu J Kroenke MA Arlehamn CL Human circulating PD-1+CXCR3-CXCR5+ memory Tfh cells are highly functional and correlate with broadly neutralizing HIV antibody responses. Immunity (2013) 39(4):758–69. 10.1016/j.immuni.2013.08.031 24035365 PMC3996844

[42] Morita R Schmitt N Bentebibel SE Ranganathan R Bourdery L Zurawski G Human blood CXCR5(+)CD4(+) T cells are counterparts of T follicular cells and contain specific subsets that differentially support antibody secretion. Immunity (2011) 34(1):108–21. 10.1016/j.immuni.2011.01.009 21215658 PMC3046815

[43] Pallikkuth S Parmigiani A Silva SY George VK Fischl M Pahwa R Impaired peripheral blood T-follicular helper cell function in HIV-infected nonresponders to the 2009 H1N1/09 vaccine. Blood (2012) 120(5):985–93. 10.1182/blood-2011-12-396648 22692510 PMC3412336

[44] Sage PT Alvarez D Godec J von Andrian UH Sharpe AH. Circulating T follicular regulatory and helper cells have memory-like properties. J Clin Invest (2014) 124(12):5191–204. 10.1172/JCI76861 25347469 PMC4348955

[45] George VK Pallikkuth S Parmigiani A Alcaide M Fischl M Arheart KL HIV infection worsens age-associated defects in antibody responses to influenza vaccine. J Infect Dis (2015) 211(12):1959–68. 10.1093/infdis/jiu840 25556252 PMC4836723

[46] Macleod MK David A McKee AS Crawford F Kappler JW Marrack P. Memory CD4 T cells that express CXCR5 provide accelerated help to B cells. J Immunol (2011) 186(5):2889–96. 10.4049/jimmunol.1002955 21270407 PMC3069687

[47] Rasheed AU Rahn HP Sallusto F Lipp M Muller G. Follicular B helper T cell activity is confined to CXCR5(hi)ICOS(hi) CD4 T cells and is independent of CD57 expression. Eur J Immunol (2006) 36(7):1892–903. 10.1002/eji.200636136 16791882

[48] Schultz BT Teigler JE Pissani F Oster AF Kranias G Alter G Circulating HIV-specific interleukin-21(+)CD4(+) T cells represent peripheral Tfh cells with antigen-dependent helper functions. Immunity (2016) 44(1):167–78. 10.1016/j.immuni.2015.12.011 26795249

[49] Akiba H Takeda K Kojima Y Usui Y Harada N Yamazaki T The role of ICOS in the CXCR5+ follicular B helper T cell maintenance in vivo. J Immunol (2005) 175(4):2340–8. 10.4049/jimmunol.175.4.2340 16081804

[50] Bentebibel SE Khurana S Schmitt N Kurup P Mueller C Obermoser G ICOS(+)PD-1(+)CXCR3(+) T follicular helper cells contribute to the generation of high-avidity antibodies following influenza vaccination. Sci Rep (2016) 6:26494 10.1038/srep26494 27231124 PMC4882544

[51] Choi YS Kageyama R Eto D Escobar TC Johnston RJ Monticelli L ICOS receptor instructs T follicular helper cell versus effector cell differentiation via induction of the transcriptional repressor Bcl6. Immunity (2011) 34(6):932–46. 10.1016/j.immuni.2011.03.023 21636296 PMC3124577

[52] Nicholas KJ Flaherty DK Smith RM Sather DN Kalams SA. Chronic HIV-1 infection impairs superantigen-induced activation of peripheral CD4+CXCR5+PD-1+ cells, with relative preservation of recall antigen-specific responses. J Acquir Immune Defic Syndr (2017) 74(1):72–80. 10.1097/QAI.0000000000001152 27509243 PMC5140753

[53] Hale JS Ahmed R Memory T follicular helper CD4 T cells. Front Immunol (2015) 6:16 10.3389/fimmu.2015.00016 25699040 PMC4313784

[54] Ueno H. Human circulating T follicular helper cell subsets in health and disease. J Clin Immunol (2016) 36(Suppl 1):34–9. 10.1007/s10875-016-0268-3 26984851

[55] Boritz EA Darko S Swaszek L Wolf G Wells D Wu X Multiple origins of virus persistence during natural control of HIV infection. Cell (2016) 166(4):1004–15. 10.1016/j.cell.2016.06.039 27453467 PMC4983216

[56] Fukazawa Y Lum R Okoye AA Park H Matsuda K Bae JY B cell follicle sanctuary permits persistent productive simian immunodeficiency virus infection in elite controllers. Nat Med (2015) 21(2):132–9. 10.1038/nm.3781 25599132 PMC4320022

[57] Perreau M Savoye AL De Crignis E Corpataux JM Cubas R Haddad EK Follicular helper T cells serve as the major CD4 T cell compartment for HIV-1 infection, replication, and production. J Exp Med (2013) 210(1):143–56. 10.1084/jem.20121932 23254284 PMC3549706

[58] Vinuesa CG. HIV and T follicular helper cells: a dangerous relationship. J Clin Invest (2012) 122(9):3059–62. 10.1172/JCI65175 22922252 PMC3428103

[59] Lindqvist M van Lunzen J Soghoian DZ Kuhl BD Ranasinghe S Kranias G Expansion of HIV-specific T follicular helper cells in chronic HIV infection. J Clin Invest (2012) 122(9):3271–80. 10.1172/JCI64314 22922259 PMC3428098

[60] Petrovas C Yamamoto T Gerner MY Boswell KL Wloka K Smith EC CD4 T follicular helper cell dynamics during SIV infection. J Clin Invest (2012) 122(9):3281–94. 10.1172/JCI63039 22922258 PMC3428091

[61] Hong JJ Amancha PK Rogers K Ansari AA Villinger F Spatial alterations between CD4(+) T follicular helper, B, and CD8(+) T cells during simian immunodeficiency virus infection: T/B cell homeostasis, activation, and potential mechanism for viral escape. J Immunol (2012) 188(7):3247–56. 10.4049/jimmunol.1103138 22387550 PMC3311732

[62] Mylvaganam GH Velu V Hong JJ Sadagopal S Kwa S Basu R Diminished viral control during simian immunodeficiency virus infection is associated with aberrant PD-1hi CD4 T cell enrichment in the lymphoid follicles of the rectal mucosa. J Immunol (2014) 193(9):4527–36. 10.4049/jimmunol.1401222 25246494 PMC4201952

[63] Hong JJ Amancha PK Rogers KA Courtney CL Havenar-Daughton C Crotty S Early lymphoid responses and germinal center formation correlate with lower viral load set points and better prognosis of simian immunodeficiency virus infection. J Immunol (2014) 193(2):797–806. 10.4049/jimmunol.1400749 24907346 PMC4084862

[64] Hong JJ Silveira E Amancha PK Byrareddy SN Gumber S Chang KT Early initiation of antiretroviral treatment postSIV infection does not resolve lymphoid tissue activation. AIDS (2017) 31(13):1819–24. 10.1097/QAD.0000000000001576 28692537 PMC5557084

[65] Boswell KL Paris R Boritz E Ambrozak D Yamamoto T Darko S Loss of circulating CD4 T cells with B cell helper function during chronic HIV infection. PLoS Pathog (2014) 10(1):e1003853. 10.1371/journal.ppat.1003853 24497824 PMC3911819

[66] Pallikkuth S Fischl MA Pahwa S. Combination antiretroviral therapy with raltegravir leads to rapid immunologic reconstitution in treatment-naive patients with chronic HIV infection. J Infect Dis (2013) 208(10):1613–23. 10.1093/infdis/jit387 23922374 PMC3805240

[67] Cubas R van Grevenynghe J Wills S Kardava L Santich BH Buckner CM Reversible reprogramming of circulating memory T follicular helper cell function during chronic HIV infection. J Immunol (2015) 195(12):5625–36. 10.4049/jimmunol.1501524 26546609 PMC4670798

[68] Cohen K Altfeld M Alter G Stamatatos L. Early preservation of CXCR5+ PD-1+ helper T cells and B cell activation predict the breadth of neutralizing antibody responses in chronic HIV-1 infection. J Virol (2014) 88(22):13310–21. 10.1128/JVI.02186-14 25210168 PMC4249103

[69] Cubas RA Mudd JC Savoye AL Perreau M van Grevenynghe J Metcalf T Inadequate T follicular cell help impairs B cell immunity during HIV infection. Nat Med (2013) 19(4):494–9. 10.1038/nm.3109 23475201 PMC3843317

[70] Moir S Malaspina A Ogwaro KM Donoghue ET Hallahan CW Ehler LA HIV-1 induces phenotypic and functional perturbations of B cells in chronically infected individuals. Proc Natl Acad Sci U S A (2001) 98(18):10362–7. 10.1073/pnas.181347898 11504927 PMC56966

[71] Moir S Fauci AS B cells in HIV infection and disease. Nat Rev Immunol (2009) 9(4):235–45. 10.1038/nri2524 19319142 PMC2779527

[72] Moir S Fauci AS. Pathogenic mechanisms of B-lymphocyte dysfunction in HIV disease. J Allergy Clin Immunol (2008) 122(1):12–9; quiz 20–1. 10.1016/j.jaci.2008.04.034 18547629 PMC2708937

[73] Moir S Chun TW Fauci AS. Pathogenic mechanisms of HIV disease. Annu Rev Pathol (2011) 6:223–48. 10.1146/annurev-pathol-011110-130254 21034222

[74] Malaspina A Moir S Kottilil S Hallahan CW Ehler LA Liu S Deleterious effect of HIV-1 plasma viremia on B cell costimulatory function. J Immunol (2003) 170(12):5965–72. 10.4049/jimmunol.170.12.5965 12794123

[75] Moir S Ho J Malaspina A Wang W DiPoto AC O’Shea MA Evidence for HIV-associated B cell exhaustion in a dysfunctional memory B cell compartment in HIV-infected viremic individuals. J Exp Med (2008) 205(8):1797–805. 10.1084/jem.20072683 18625747 PMC2525604

[76] Pensieroso S Cagigi A Palma P Nilsson A Capponi C Freda E Timing of HAART defines the integrity of memory B cells and the longevity of humoral responses in HIV-1 vertically-infected children. Proc Natl Acad Sci U S A (2009) 106(19):7939–44. 10.1073/pnas.0901702106 19416836 PMC2683072

[77] Amu S Lavy-Shahaf G Cagigi A Hejdeman B Nozza S Lopalco L Frequency and phenotype of B cell subpopulations in young and aged HIV-1 infected patients receiving ART. Retrovirology (2014) 11:76. 10.1186/s12977-014-0076-x 25213015 PMC4172851

[78] Pensieroso S Galli L Nozza S Ruffin N Castagna A Tambussi G B-cell subset alterations and correlated factors in HIV-1 infection. AIDS (2013) 27(8):1209–17. 10.1097/QAD.0b013e32835edc47 23343911

[79] Cagigi A Rinaldi S Di Martino A Manno EC Zangari P Aquilani A Premature immune senescence during HIV-1 vertical infection relates with response to influenza vaccination. J Allergy Clin Immunol (2014) 133(2):592–4. 10.1016/j.jaci.2013.10.003 24290278

[80] Keim C Kazadi D Rothschild G Basu U. Regulation of AID, the B-cell genome mutator. Genes Dev (2013) 27(1):1–17. 10.1101/gad.200014.112 23307864 PMC3553278

[81] Klein U Dalla-Favera R. Germinal centres: role in B-cell physiology and malignancy. Nat Rev Immunol (2008) 8(1):22–33. 10.1038/nri2217 18097447

[82] Allen CD Okada T Cyster JG. Germinal-center organization and cellular dynamics. Immunity (2007) 27(2):190–202. 10.1016/j.immuni.2007.07.009 17723214 PMC2242846

[83] Victora GD Nussenzweig MC. Germinal centers. Annu Rev Immunol (2012) 30:429–57. 10.1146/annurev-immunol-020711-075032 22224772

[84] Hart M Steel A Clark SA Moyle G Nelson M Henderson DC Loss of discrete memory B cell subsets is associated with impaired immunization responses in HIV-1 infection and may be a risk factor for invasive pneumococcal disease. J Immunol (2007) 178(12):8212–20. 10.4049/jimmunol.178.12.8212 17548660

[85] Siberry GK Patel K Bellini WJ Karalius B Purswani MU Burchett SK Immunity to measles, mumps, and rubella in US children with perinatal HIV infection or perinatal HIV exposure without infection. Clin Infect Dis (2015) 61(6):988–95. 10.1093/cid/civ440 26060291 PMC4551008

[86] Schmitt N Bentebibel SE Ueno H. Phenotype and functions of memory Tfh cells in human blood. Trends Immunol (2014) 35(9):436–42. 10.1016/j.it.2014.06.002 24998903 PMC4152409

[87] Herati RS Reuter MA Dolfi DV Mansfield KD Aung H Badwan OZ Circulating CXCR5+PD-1+ response predicts influenza vaccine antibody responses in young adults but not elderly adults. J Immunol (2014) 193(7):3528–37. 10.4049/jimmunol.1302503 25172499 PMC4170011

[88] Pallikkuth S Kanthikeel SP Silva SY Fischl M Pahwa R Pahwa S. Innate immune defects correlate with failure of antibody responses to H1N1/09 vaccine in HIV-infected patients. J Allergy Clin Immunol (2011) 128(6):1279–85. 10.1016/j.jaci.2011.05.033 21752440 PMC3229646

[89] Pallikkuth S Pilakka Kanthikeel S Silva SY Fischl M Pahwa R Pahwa S. Upregulation of IL-21 receptor on B cells and IL-21 secretion distinguishes novel 2009 H1N1 vaccine responders from nonresponders among HIV-infected persons on combination antiretroviral therapy. J Immunol (2011) 186(11):6173–81. 10.4049/jimmunol.1100264 21531891 PMC3170914

[90] Cotugno N de Armas LR Pallikkuth S Pan L Rinaldi S Sanchez MC Perturbation of B cell gene-expression persists in HIV infected children despite effective ART and predicts H1N1 response. Front Immunol (2017) 8:1083 10.3389/fimmu.2017.01083 28955330 PMC5600985

[91] de Armas LR Cotugno N Pallikkuth S Pan L Rinaldi S Sanchez MC Induction of IL21 in peripheral T follicular helper cells is an indicator of influenza vaccine response in a previously vaccinated HIV-infected pediatric cohort. J Immunol (2017) 198(5):1995–2005. 10.4049/jimmunol.1601425 28130496 PMC5322168

[92] Johnston RJ Choi YS Diamond JA Yang JA Crotty S. STAT5 is a potent negative regulator of TFH cell differentiation. J Exp Med (2012) 209(2):243–50. 10.1084/jem.20111174 22271576 PMC3281266

[93] Heit A Schmitz F Gerdts S Flach B Moore MS Perkins JA Vaccination establishes clonal relatives of germinal center T cells in the blood of humans. J Exp Med (2017) 214(7):2139–52. 10.1084/jem.20161794 28637884 PMC5502430

[94] Herati RS Muselman A Vella L Bengsch B Parkhouse K Del Alcazar D Successive annual influenza vaccination induces a recurrent oligoclonotypic memory response in circulating T follicular helper cells. Sci Immunol (2017) 2(8):eaag2152 10.1126/sciimmunol.aag2152 28620653 PMC5469419

[95] Bentebibel SE Lopez S Obermoser G Schmitt N Mueller C Harrod C Induction of ICOS+CXCR3+CXCR5+ TH cells correlates with antibody responses to influenza vaccination. Sci Transl Med (2013) 5(176):176ra32. 10.1126/scitranslmed.3005191 23486778 PMC3621097

[96] Farooq F Beck K Paolino KM Phillips R Waters NC Regules JA Circulating follicular T helper cells and cytokine profile in humans following vaccination with the rVSV-ZEBOV Ebola vaccine. Sci Rep (2016) 6:27944. 10.1038/srep27944 27323685 PMC4914957

[97] Alcaide ML Parmigiani A Pallikkuth S Roach M Freguja R Della Negra M Immune activation in HIV-infected aging women on antiretrovirals – implications for age-associated comorbidities: a cross-sectional pilot study. PLoS One (2013) 8(5):e63804 10.1371/journal.pone.0063804 23724003 PMC3665816

[98] Parmigiani A Alcaide ML Freguja R Pallikkuth S Frasca D Fischl MA Impaired antibody response to influenza vaccine in HIV-infected and uninfected aging women is associated with immune activation and inflammation. PLoS One (2013) 8(11):e79816. 10.1371/journal.pone.0079816 24236161 PMC3827419

[99] de Armas LR Pallikkuth S George V Rinaldi S Pahwa R Arheart KL Re-evaluation of immune activation in the era of cART and an aging HIV-infected population. JCI Insight (2017) 2(20):e95726 10.1172/jci.insight.95726 PMC5846952 29046481

[100] Rinaldi S Pallikkuth S George VK de Armas LR Pahwa R Sanchez CM Paradoxical aging in HIV: immune senescence of B Cells is most prominent in young age. Aging (2017) 9(4):1307–25. 10.18632/aging.101229 28448963 PMC5425129

[101] Caraux A Klein B Paiva B Bret C Schmitz A Fuhler GM Circulating human B and plasma cells. Age-associated changes in counts and detailed characterization of circulating normal CD138- and CD138+ plasma cells. Haematologica (2010) 95(6):1016–20. 10.3324/haematol.2009.018689 20081059 PMC2878802

[102] Klein SL Jedlicka A Pekosz A. The Xs and Y of immune responses to viral vaccines. Lancet Infect Dis (2010) 10(5):338–49. 10.1016/S1473-3099(10)70049-9 20417416 PMC6467501

[103] Haralambieva IH Ovsyannikova IG Kennedy RB Larrabee BR Shane Pankratz V Poland GA. Race and sex-based differences in cytokine immune responses to smallpox vaccine in healthy individuals. Hum Immunol (2013) 74(10):1263–6. 10.1016/j.humimm.2013.06.031 23806267 PMC4170575

[104] Gardner EM Gonzalez EW Nogusa S Murasko DM. Age-related changes in the immune response to influenza vaccination in a racially diverse, healthy elderly population. Vaccine (2006) 24(10):1609–14. 10.1016/j.vaccine.2005.09.058 16260072

[105] Christy C Pichichero ME Reed GF Decker MD Anderson EL Rennels MB Effect of gender, race, and parental education on immunogenicity and reported reactogenicity of acellular and whole-cell pertussis vaccines. Pediatrics (1995) 96(3 Pt 2):584–7. 7659481

[106] Klein SL Marriott I Fish EN. Sex-based differences in immune function and responses to vaccination. Trans R Soc Trop Med Hyg (2015) 109(1):9–15. 10.1093/trstmh/tru167 25573105 PMC4447843

[107] Fink AL Klein SL. Sex and gender impact immune responses to vaccines among the elderly. Physiology (2015) 30(6):408–16. 10.1152/physiol.00035.2015 26525340 PMC4630198

[108] Falsey AR Treanor JJ Tornieporth N Capellan J Gorse GJ. Randomized, double-blind controlled phase 3 trial comparing the immunogenicity of high-dose and standard-dose influenza vaccine in adults 65 years of age and older. J Infect Dis (2009) 200(2):172–80. 10.1086/599790 19508159

[109] Furman D Hejblum BP Simon N Jojic V Dekker CL Thiebaut R Systems analysis of sex differences reveals an immunosuppressive role for testosterone in the response to influenza vaccination. Proc Natl Acad Sci U S A (2014) 111(2):869–74. 10.1073/pnas.1321060111 24367114 PMC3896147

[110] Furman D. Sexual dimorphism in immunity: improving our understanding of vaccine immune responses in men. Expert Rev Vaccines (2015) 14(3):461–71. 10.1586/14760584.2015.966694 25278153

[111] Merani S Pawelec G Kuchel GA McElhaney JE. Impact of aging and cytomegalovirus on immunological response to influenza vaccination and infection. Front Immunol (2017) 8:784. 10.3389/fimmu.2017.00784 28769922 PMC5512344

[112] Derhovanessian E Maier AB Hahnel K McElhaney JE Slagboom EP Pawelec G. Latent infection with cytomegalovirus is associated with poor memory CD4 responses to influenza A core proteins in the elderly. J Immunol (2014) 193(7):3624–31. 10.4049/jimmunol.1303361 25187662

[113] Derhovanessian E Theeten H Hahnel K Van Damme P Cools N Pawelec G. Cytomegalovirus-associated accumulation of late-differentiated CD4 T-cells correlates with poor humoral response to influenza vaccination. Vaccine (2013) 31(4):685–90. 10.1016/j.vaccine.2012.11.041 23196209

[114] den Elzen WP Vossen AC Cools HJ Westendorp RG Kroes AC Gussekloo J. Cytomegalovirus infection and responsiveness to influenza vaccination in elderly residents of long-term care facilities. Vaccine (2011) 29(29–30):4869–74. 10.1016/j.vaccine.2011.03.086 21497631

[115] Channappanavar R Twardy BS Krishna P Suvas S. Advancing age leads to predominance of inhibitory receptor expressing CD4 T cells. Mech Ageing Dev (2009) 130(10):709–12. 10.1016/j.mad.2009.08.006 19715717

[116] Shimada Y Hayashi M Nagasaka Y Ohno-Iwashita Y Inomata M. Age-associated up-regulation of a negative co-stimulatory receptor PD-1 in mouse CD4+ T cells. Exp Gerontol (2009) 44(8):517–22. 10.1016/j.exger.2009.05.003 19457448

[117] Kahan SM Wherry EJ Zajac AJ T cell exhaustion during persistent viral infections. Virology (2015) 47(9–480):180–93. 10.1016/j.virol.2014.12.033 PMC4424083 25620767

[118] McKinney EF Smith KG. T-cell exhaustion: understanding the interface of chronic viral and autoinflammatory diseases. Immunol Cell Biol (2016) 94(10):935–42. 10.1038/icb.2016.81 27577866

[119] Ye B Liu X Li X Kong H Tian L Chen Y. T-cell exhaustion in chronic hepatitis B infection: current knowledge and clinical significance. Cell Death Dis (2015) 6:e1694. 10.1038/cddis.2015.42 25789969 PMC4385920

[120] Zehn D Wherry EJ. Immune memory and exhaustion: clinically relevant lessons from the LCMV model. Adv Exp Med Biol (2015) 850:137–52. 10.1007/978-3-319-15774-0_10 26324351

[121] Rasmussen TA Tolstrup M Sogaard OS Reversal of latency as part of a cure for HIV-1. Trends Microbiol (2016) 24(2):90–7. 10.1016/j.tim.2015.11.003 26690612

